# Machine learning and 4D-LFQ quantitative proteomic analysis explore the molecular mechanism of kidney stone formation

**DOI:** 10.1016/j.heliyon.2024.e34405

**Published:** 2024-07-10

**Authors:** Ziqi He, Jiawei Zhou, Caitong Dong, Chao Song, Wenbiao Liao, Yunhe Xiong, Sixing Yang

**Affiliations:** Department of Urology, Renmin Hospital of Wuhan University, Wuhan, 430060, Hubei Province, People's Republic of China

**Keywords:** Nephrolithiasis, Machine learning, 4D-LFQ, Calcium oxalate, HK-2 cells, Proteomics, Parallel reaction monitoring

## Abstract

**Background:**

Nephrolithiasis, a common and chronic urological condition, exerts significant pressure on both the general public and society as a whole. The precise mechanisms of nephrolith formation remain inadequately comprehended. Nevertheless, the utilization of proteomics methods has not been employed to examine the development of renal calculi in order to efficiently hinder and manage the creation and reappearance of nephrolith. Nowadays, with the rapid development of proteomics techniques, more efficient and more accurate proteomics technique is utilized to uncover the mechanisms underlying diseases. The objective of this study was to investigate the possible alterations of HK-2 cells when exposed to varying amounts of calcium oxalate (CaOx). The aim was to understand the precise development of stone formation and recurrence, in order to find effective preventive and treatment methods.

**Methods:**

To provide a complete view of the proteins involved in the development of nephrolithiasis, we utilized an innovative proteomics method called 4D-LFQ proteomic quantitative techniques. HK-2 cells were selected as our experimental subjects. Three groups (n = 3) of HK-2 cells were treated with intervention solutions containing 0 (negative control, NC), 1 mM, and 2 mM CaOx, respectively. For the proteins that showed differential expression, various analyses were conducted including examination of Gene Ontology (GO), Clusters of Orthologous Groups of proteins (KOG), Kyoto Encyclopedia of Genes and Genomes (KEGG) pathway, enrichment analysis of protein domains, and hierarchical clustering analysis. The STRING database was used to identify the interaction network of the chosen proteins. Candidate proteins were validated using parallel reaction monitoring (PRM) in the end.

**Results:**

All three groups verified the repeatability of samples. According to the results of 4D-LFQ proteomic quantitative analysis, there were 120, 262, and 81 differentially expressed proteins (DEPs) in the 1 mM-VS-NC, 2 mM-VS-NC, and 2 mM-VS-1mM conditions, respectively. According to GO annotation, the functional enrichment analysis indicates that the differentially expressed proteins (DEPs) were notably enriched in promoting cell migration and the extracellular matrix, among other functions. Analysis of enrichment, based on the KEGG pathway, revealed significant enrichment of DEPs in complement and coagulation cascades, as well as in ECM-receptor (extracellular matrix-receptor) interaction and other related pathways. 14 DEPs of great interest were selected as candidate proteins, including FN1, TFRC, ITGA3, FBN1, HYOU1, SPP1, HSPA5, COL6A1, MANF, HIP1R, JUP, AXL, CTNNB1 and DSG2.The data from PRM demonstrated the variation trend of 14 DEPs was identical as 4D-LFQ proteomic quantitative analysis.

**Conclusion:**

Proteomics studies of CaOx-induced HK-2 cells using 4D-LFQ proteomic quantitative analysis and PRM may help to provide crucial potential target proteins and signaling pathways for elucidating the mechanism of nephrolithiasis and better treating nephrolithiasis.

## 1 Introduction

Nephrolithiasis, commonly termed kidney stone, is a prevalent urinary condition [1]. The high prevalence and recurrence rate of nephrolithiasis, coupled with substantial treatment expenses, significantly impacts both patients and society [2,3]. Currently, the increasing prevalence of this condition, associated with intense lower back discomfort and complications like pyelonephritis and Acute Kidney Injury (AKI), has garnered significant attention globally [4]. In the absence of effective measures, the recurrence rate for patients having undergone stone removal therapy has been estimated to be around 35%–50 % [5]. Despite extensive research, the precise mechanisms underlying nephrolithiasis remain elusive, considerably impeding the development of targeted therapies. Therefore, additional research to elucidate the exact pathogenic processes involved in stone formation is essential for the effective prevention and treatment of stone formation and recurrence.

The crucial role of calcium oxalate (CaOx) in the formation of the majority of urinary stones has been widely acknowledged. A minority of stones are composed of calcium phosphate (CaP, also known as apatite) or a combination of CaOx and CaP [6]. Numerous studies have demonstrated a close association between the formation of kidney stones and oxidative-stress-induced damage in renal tubular epithelial cells caused by hyperoxaluria or CaOx crystals [6,7]. Furthermore, the interaction between the cells lining the renal tubules and CaOx ions is posited as a critical factor for the formation of CaOx stones [5]. Damage to tubular epithelial cells caused by the excessive production of ROS (Reactive Oxygen Species) has been corroborated by extensive animal experiments [6]. The presence of abnormal calcium and oxalate levels, overproduction of intracellular ROS, and a decrease in antioxidant capacity lead to oxidative stress, ERS (Endoplasmic Reticulum Stress), and autophagy dysfunction, among other outcomes [8]. Recent research accentuated the significance of HK-2 cell-induced damage in the proximal tubule in the renal cortex, a critical process during the initial development of kidney stones [9]. Under persistent high CaOx concentrations, the HK-2 cells generate excessive ROS and undergo oxidative stress, leading to cell degeneration, apoptosis, basement membrane exposure, and other injuries. Consequently, crystal nucleation, aggregation, and growth occur in succession [10]. Thus, HK-2 cells were selected as experimental subjects for the current study instead of other renal tubular epithelial cells, predominantly utilized in previous research, due to the significant role of the proximal tubule in calcium and oxalate reabsorption [11].

Recently, proteomics has emerged as an increasingly promising tool for elucidating the role of proteins in diseases, including underlying mechanisms, as it enables the simultaneous detection of both known and unknown proteins [12]. To effectively prevent and treat stone formation and recurrence, a study using the 4D-LFQ quantitative proteomic analysis technique was undertaken. This method, offering exceptional precision and robust capabilities, facilitated the in-depth examination of protein characteristics in HK-2 cells when exposed to varying CaOx doses. The objective of this study was to acquire a comprehensive understanding of the precise pathogenesis involved in order to develop effective preventive and therapeutic strategies.

## 2 Methods and materials

### 2.1 Cells, cell culture and exposure

The Cell Bank of the Chinese Academy of Sciences generously provided HK-2 cells, which were cultured in complete DMEM/F12 (with the addition of 10 % fetal bovine serum and 100 U/ml penicillin/streptomycin as previously described) at a temperature of 37 °C in a 5 % CO2 incubator. Upon reaching a cell density of 80 %, the complete DMEM/F12 should be substituted with serum-free DMEM/F12. The serum-free DMEM/F12 should be prepared as three different solutions: 0 (negative control, NC), 1 mM, and 2 mM CaOx intervention. Consequently, the cells should be divided into three groups (n = 9) and incubated for 24 h before analysis.

### 2.2 Protein extraction and trypsin digestion

The extraction and digestion procedures were adhered to in accordance with the method that was published [[13], [14], [15], [16]]. Samples were extracted from a cryogenic refrigerator at a temperature of −80 °C. Subsequently, we subjected them to three rounds of sonication on ice using a high-powered ultrasonic processor (Scientz) in a lysis buffer with four times the volume (8 M urea, 1 % Protease Inhibitor Cocktail). Centrifugation at 12,000 g at 4 °C for 10 min effectively eliminated the remaining debris. Afterwards, the liquid above was gathered and moved to fresh tubes for centrifugation. In the end, we ascertained the protein concentration using a BCA kit according to the guidelines provided by the manufacturer.

To ensure equal quantities of enzymolysis for each group of proteins, we utilized a lysis buffer to maintain consistent volume. Afterwards, we introduced a single volume of chilled acetone into the sample, followed by adding four volumes of chilled acetone after vortex mixing, and subsequently allowing them to precipitate at −20 °C for a duration of 2 h. After centrifuging the mixture at a speed of 4500 g for 5 min, the liquid portion was discarded and the solid residue was rinsed two times with chilled acetone. After the sediment was dried, we added 200 mM triethyl ammonium bicarbonate (TEAB) into the centrifugal tubes and sonicated the sediment. Afterwards, trypsin was included at a trypsin-to-protein mass ratio of 1:50 for overnight digestion. In the end, the protein solution was treated with 5 mM dithiothreitol at a temperature of 56 °C for 30 min and then exposed to 11 mM iodoacetamide for 15 min at room temperature in the absence of light.

### 2.3 LC-MS/MS analysis

The LC-MS/MS analysis procedures were adhered to in accordance with the method that was published [17,18]. Following trypsin cleavage, the tryptic peptides were dissolved in a solution of 0.1 % formic acid (solvent A) and then loaded onto a reversed-phase analytical column that was 15 cm in length and had an inner diameter of 75 μm. The slope consisted of a rise from 2 % to 5 % solvent B (0.1 % formic acid in 98 % acetonitrile) within 1 min, followed by an increase from 5 % to 27 % over a period of 75 min, then a further increase from 27 % to 35 % in just 6 min. Finally, it reached 85 % within 4 min, while maintaining a constant flow rate of 300.00 nL/min using an EASY-nLC 1000 UPLC system. The peptides underwent NSI source and were then analyzed using tandem mass spectrometry (MS/MS) in Q ExactiveTM Plus (Thermo) connected online to the UPLC. A voltage of 2.0 kV was applied for electrospray. The mass-to-charge ratio scan range spanned from 100 to 1700 during the full scan, and the Orbitrap detected complete peptides with a resolution of 70,000. Following the selection of peptides, MS/MS was performed using an NCE setting of 28, and the fragments were subsequently detected in the Orbitrap with a resolution of 17,500. An algorithm that relied on data performed a sequence of one MS scan followed by 10 MS/MS scans, implementing a dynamic exclusion of 30.0s. The AGC was adjusted to 50,000 using automatic gain control (AGC). The initial mass was established at 100 *m*/*z*.

### 2.4 Databases search and bioinformatic analysis

Maxquant search engine (v.1.6.15.0) was used to process the resulting MS/MS data. To decrease the False Positive Rate (FDR), tandem mass spectra were compared to a combination of the human uniprot database and the reverse decoy database during the search process. The cleavage enzyme specified was Trypsin/P, with a tolerance of up to 2 missing cleavages. In the First search, the mass tolerance for precursor ions was established at 20.0 ppm, while in the Main search it was set at 20 ppm. Additionally, the mass tolerance for fragment ions was defined as 0.02 Da. The FDR was reduced to less than 1 % and the minimum score for modified peptides was set to be greater than 40.

We conducted a study where we compared the MS/MS data obtained from three different groups: the NC group, the 1 mM CaOx intervention group, and the 2 mM CaOx intervention group. This resulted in the formation of three research objects, namely 1 mM-VS-NC, 2 mM-VS-NC, and 2 mM-VS-1mM. The focus was on the fold-change in protein expression between the intervention with 1 mM CaOx, intervention with 2 mM CaOx, and the NC group. Significant expression was attributed to proteins with a fold-change of ≥1.50 or ≤0.67 and a p-value <0.05 in 1 mM-VS-NC, 2 mM-VS-NC, and 2 mM-VS-1mM. To gain a comprehensive understanding of the proteins identified and quantified in the study, we extensively examined the function and characteristics of these proteins. This was done by utilizing WoLF PSORT for subcellular localization, UniProt-GOA database for GO annotation (http://www.ebi.ac.uk/GOA/), and KOG (Clusters of Orthologous Groups of proteins) functional classification for analysis. Moreover, in order to determine if there are significant enrichment trends in certain functional types for differential expression proteins (DEPs), we performed functional annotation enrichments of DEPs using domain annotation from the InterPro database (http://www.ebi.ac.uk/interpro/), GO annotation, and KEGG (Kyoto Encyclopedia of Genes and Genomes) pathway annotation. The substantial enhancement was determined as a corrected p-value less than 0.05 in a two-tailed Fisher's exact test. The DEPs were subjected to hierarchical clustering analysis using the 'heatmap.2′ function from the R package 'gplots'. In the end, we utilized the STRING database (https://cn.string-db.org/) to construct a network of protein-protein interactions for the chosen proteins.

### 2.5 Construction and validation of filtering hub genes using machine learning

In PD, the Random Forest Algorithm utilizes Recursive feature elimination (RFE) as a supervised machine learning technique to sequence genes linked to copper poisoning. R software was used to construct a random forest (RF) tree model, support vector machine (SVM) learning model, extreme gradient boosting (XGBoost) model, and general linear model (GLM) using the clinical traits and common trait genes obtained above. Distinctive genes were identified after calculating the prediction performance using ten-fold cross-validation and determining their relative relevance to be greater than 0.25.SVM, a compact learning method, effectively bypasses the traditional process of moving from induction to deduction. It accomplishes efficient 'reverse reasoning' by extrapolating from sample training to prediction, simplifying intricate classification and regression problems. XGBoost is an exemplary integrated boosting algorithm that tackles the problem of overfitting in gradient boosting models. The GLM is an advancement of the traditional linear model, which utilizes a nonlinear link function to better handle and gather data that is not normally distributed. Before constructing a diagnostic model with the mentioned four methods, the data samples were first subjected to residual analysis to illustrate the reverse cumulative distribution of residuals for the four approaches.

### 2.6 Parallel reaction monitoring (PRM) Verifying target proteins

The extraction and digestion procedures were carried out in a manner identical to that of the previous investigation. Next, the tryptic peptides were dissolved in a solution of 0.1 % formic acid (solvent A) and subsequently injected into a custom-made reversed-phase analytical column. The slope consisted of a rise from 8 % to 30 % solvent B (0.1 % formic acid in 90 % acetonitrile) within a duration of 16 min, followed by an increase from 30 % to 40 % in 6 min. Subsequently, it ascended to 80 % in 4 min and remained steady at 80 % for the final 4 min. Throughout this process, a consistent flow rate of 500 nL/min was maintained using an EASY-nLC 1000 UPLC system.

The peptides underwent NSI source and were then analyzed using tandem mass spectrometry (MS/MS) in Q ExactiveTM Plus (Thermo) connected online to the UPLC. A voltage of 2.1 kV was applied for electrospray. For the full scan, the range of *m*/*z* scan was from 455 to 1000, and the Orbitrap detected intact peptides with a resolution of 70,000. Following the selection of peptides for MS/MS, an NCE setting of 27 was used, and the fragments were subsequently detected in the Orbitrap with a resolution of 17,500. A procedure that was not influenced by data, which involved alternating between a single MS scan and 20 MS/MS scans. The AGC was configured to 3E6 for full MS and 1E5 for MS/MS. For full MS, the maximum IT was established at 50 ms, while for MS/MS, it was set to auto. The MS/MS isolation window was configured to be 1.6 *m*/*z*.Skyline (v.3.6) was utilized to process the obtained MS data.

### 2.7 Statistical analysis

Statistical analysis was conducted using SPSS 22.0 software. Quantitative data were analyzed using one-way ANOVA, while the rank sum test was conducted for those with non-uniform variance. The results were presented as the average plus or minus the standard error of the mean. A significance level of p < 0.05 was established as the threshold for determining statistical difference.

## 3 Results

### 3.1 The repeatability of samples test

**Fig. 1:**
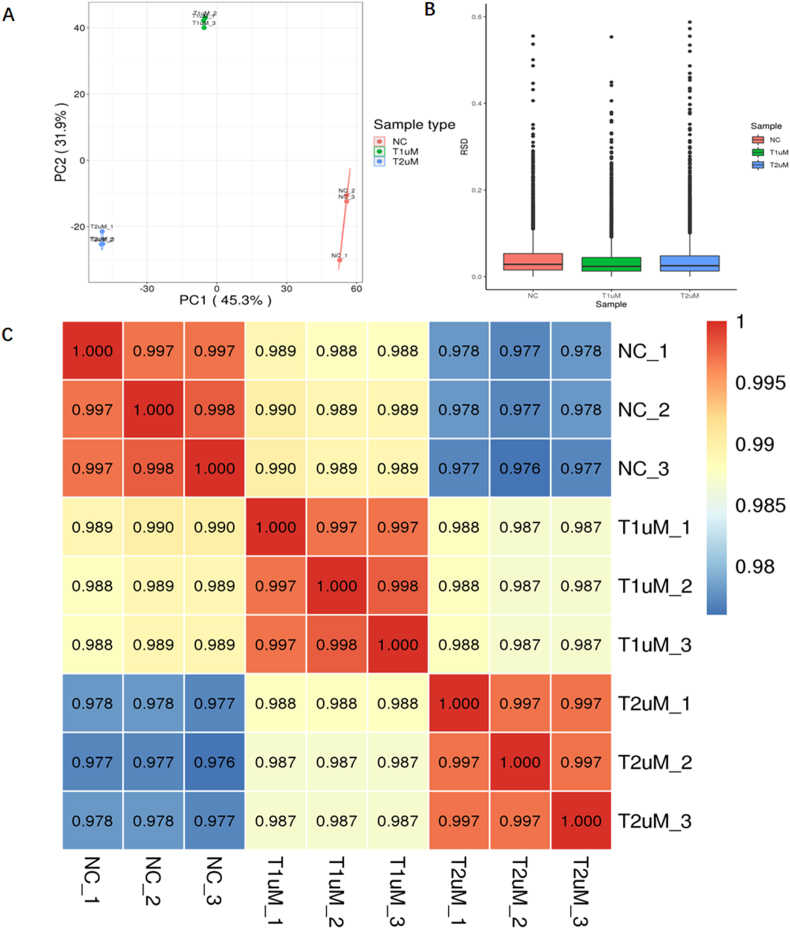
The repeatability of samples test. (A) PCA (Principal Component Analysis), the closer the degree of aggregation among repeated samples is, the better the repeatability of protein quantification is. (B) The boxplot indicates the RSD (Relative Standard Deviation) of the value of protein quantification among repeated samples. When the overall RSD is smaller, the repeatability is greater. (C) Pearson Correlation Coefficient is a value that measures the degree of linear correlation between two sets of data. When approaching −1, it represents negative correlation; when approaching 1, it represents positive correlation; when approaching 0, it represents no correlation.

An array of tests were conducted on the samples from the NC group (n = 3), the 1 mM CaOx intervention group (n = 3), and the 2 mM CaOx intervention group (n = 3) to ascertain the statistical consistency of repeated samples. Three statistical analysis techniques were employed to assess the repeatability: PCA (Principal Component Analysis), RSD (Relative Standard Deviation), and Pearson Correlation Coefficient. PCA revealed tight clustering for the three samples from each group, as evidenced by the results (Fig. 1A). Additionally, the RSD levels consistently remained low, and the Pearson Correlation Coefficient closely approached unity in all three groups (Fig. 1B and C). In summary, satisfactory consistency in protein measurement was demonstrated across the three categories by these evaluations.

### 3.2 The overview of protein identification

**Fig. 2:**
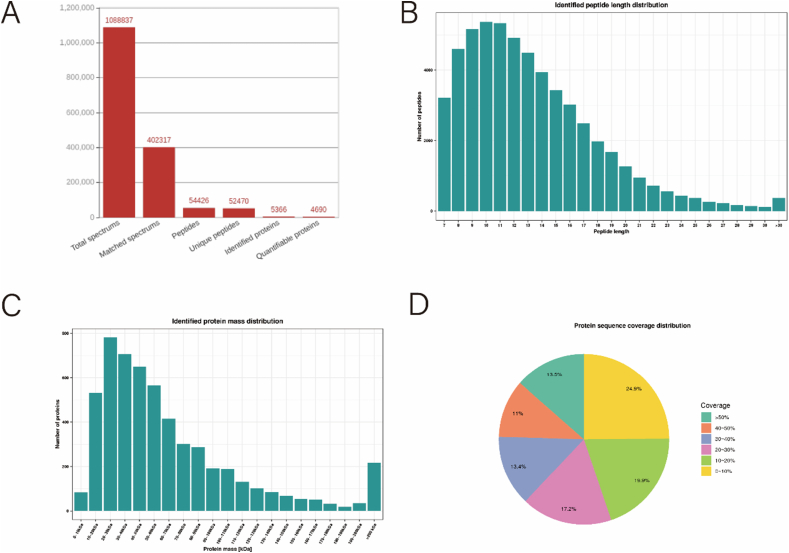
Protein identification. (A) Basic statistics of mass spectrometry data. (B) The distribution of the length of all identified peptide fragments. Peptides with less than 5 amino acids do not yield effective sequence identification due to too few fragment ions being produced. Peptides with more than 20 amino acids are not suitable for HCD fragmentation due to their high mass and charge. (C) Proteins above 10 kDa were evenly distributed and there was no obvious molecular weight bias for proteins above 10 KDa during sample preparation. (D) The distribution of protein sequence coverage.

Mass spectrometry yielded 108,8837 secondary spectra in our investigation. Post analysis against the human Uniprot database, 40,2317 spectra were deemed utilizable, translating to a spectral utilization rate of 35.9 %. A total of 54,426 peptides were detected by examining the spectrogram, including 52,470 unique peptides. Moreover, 5366 proteins were identified, with 4690 categorized as measurable proteins (Fig. 2A). The majority of peptide fragments comprised amino acids ranging from 7 to 20, and the identified protein mass was primarily within the 10–100 kDa range (Fig. 2B and C), aligning with the anticipated outcomes of trypsin digestion and HCD fragmentation. The mass spectrometry analysis successfully met the quality control criteria for the distribution of identified peptide fragments. Furthermore, over 75.1 % of the protein sequences achieved a coverage exceeding 10 % (Fig. 2D), signifying the successful preparation of the samples and their suitability for subsequent analysis.

### 3.3 Proteome alterations of HK-2 cells in response to differential CaOx doses

**Fig. 3:**
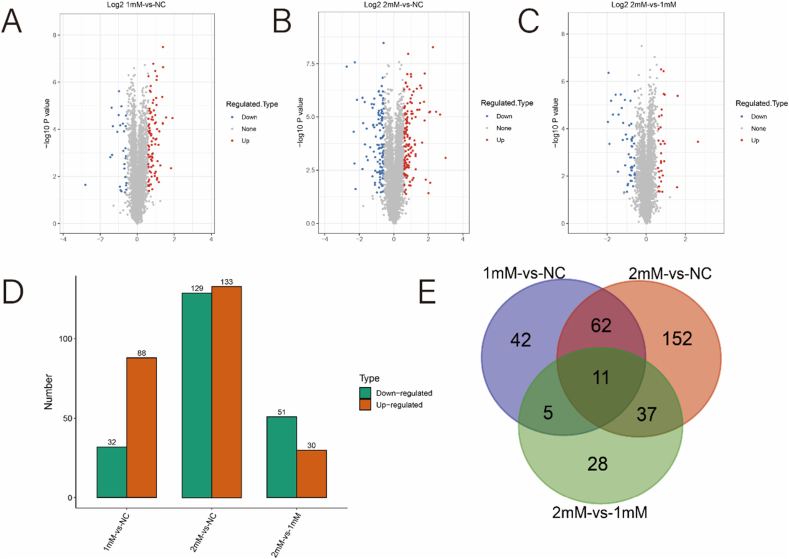
Proteome alterations of HK-2 cells. (A) Volcano Plot of DEPs in 1 mM-VS-NC. Proteins whose p < 0.05 and fold-change ≥1.50 were marked red and proteins whose p < 0.05 and fold-change ≤0.67 were marked blue. (B) Volcano Plot of DEPs in 2 mM-VS-NC. (C) Volcano Plot of DEPs in 2 mM-VS-1mM. (D) Number of DEPs among 1 mM-VS-NC, 2 mM-VS-NC and 2 mM-VS-1mM, respectively. (E) Venn diagram among 1 mM-VS-NC, 2 mM-VS-NC and 2 mM-VS-1mM, respectively.

From the proteins identified in LC-MS/MS, 4690 were quantifiable. The screening parameters for further bioinformatic analysis were established as a p-value <0.05 and a fold-change ≥1.50 or ≤0.67 for significantly differentially expressed proteins (DEPs). In comparison to the NC group, the 1 mM CaOx intervention group exhibited 120 DEPs, including 88 upregulated proteins (with a fold-change ≥1.50) and 32 downregulated proteins (with a fold-change ≤0.67) (Fig. 3A). In comparison to the NC group, the 2 mM CaOx intervention group exhibited 262 DEPs, with 133 proteins showing upregulated expression (fold-change ≥1.50) and 129 proteins showing downregulated expression (fold-change ≤0.67) (Fig. 3B). Meanwhile, 81 DEPs (with 30 showing upregulation and 51 showing downregulation) were identified in the 2 mM CaOx intervention group compared to the 1 mM CaOx intervention group (Fig. 3C). Fig. 3D illustrates a histogram detailing the quantity of DEPs across the three research subjects. Furthermore, the current research indicated that 73, 16, and 48 DEPs were common in the 1 mM-VS-NC and 2 mM-VS-NC, 1 mM-VS-NC and 2 mM-VS-1mM, and 2 mM-VS-NC and 2 mM-VS-1mM comparisons, respectively. Noteworthily, 11 DEPs were the same in 1 mM-VS-NC, 2 mM-VS-NC, and 2 mM-VS-1mM (Table 1). The results indicated that these proteins exhibited significant differential expression (p < 0.05) in HK-2 cells under varying CaOx concentrations. Conversely, no notable disparity (p > 0.05) was observed in the expression of 62 identical DEPs when exposed to CaOx concentrations of 1 mM and 2 mM. Fig. 3E presents a Venn diagram illustrating the interrelations among the three research subjects.

**Table 1:** 11 identical DEPs were present in 1 mM-VS-NC, 2 mM-VS-NC, and 2 mM-VS-1mM.

Protein accession	Gene name	1 mM-VS-NC fold-change	Regulated Type	2 mM-VS-NC fold-change	Regulated Type	2 mM-VS-1mM fold-change	Regulated Type
A6NHL2	TUBAL3	1.8195	Up	0.6088	Down	0.3346	Down
P00749	PLAU	3.7127	Up	6.3348	Up	1.7062	Up
P16401	H1-5	2.9229	Up	5.3985	Up	1.847	Up
P16403	H1-2	2.5956	Up	4.7561	Up	1.8323	Up
P50914	RPL14	2.0484	Up	3.4255	Up	1.6722	Up
P62273	RPS29	0.5245	Down	0.1494	Down	0.2849	Down
Q13427	PPIG	0.5892	Down	0.3483	Down	0.5911	Down
Q13751	LAMB3	2.5807	Up	4.2431	Up	1.6442	Up
Q15059	BRD3	0.3615	Down	0.2069	Down	0.5725	Down
Q9NQ84	GPRC5C	2.2686	Up	4.0989	Up	1.8068	Up
Q9Y240	CLEC11A	1.9841	Up	3.0851	Up	1.5549	Up

### 3.4 Functional classification of DEPs

**Fig. 4:**
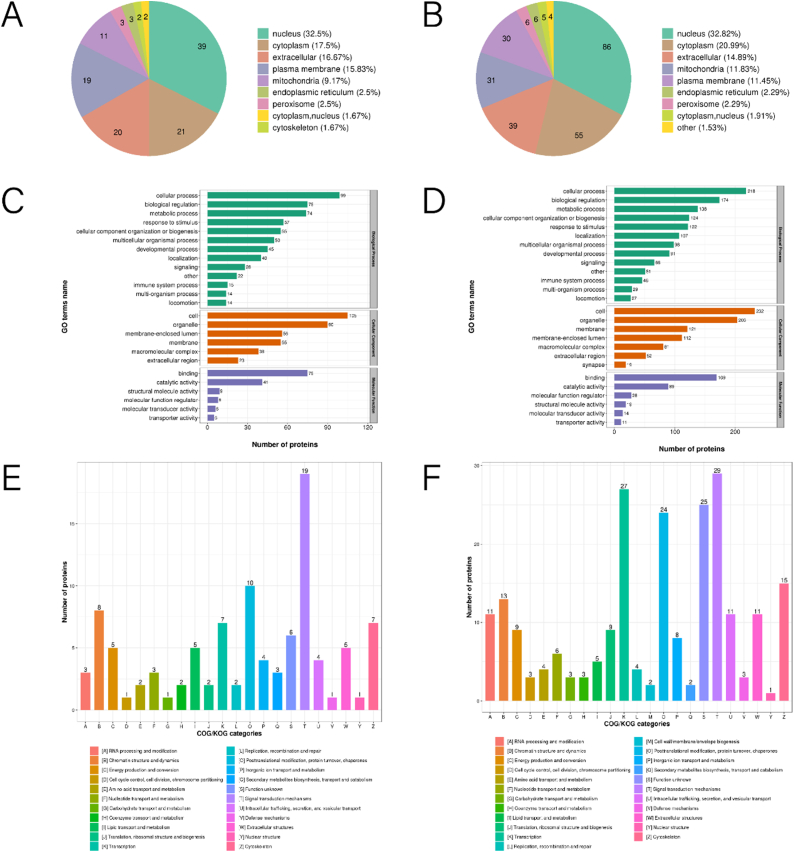
Functional classification of DEPs. (A, B) Subcellular location of DEPs in 1 mM-VS-NC and 2 mM-VS-NC, respectively. (C, D) Functional distribution of DEPs in GO annotations. (E, F) Functional classifications of DEPs based on KOG database. DEPs, differential expressed proteins.

The subcellular localization of DEPs in 1 mM-VS-NC and 2 mM-VS-NC was explored. Analysis in 1 mM-VS-NC revealed that the majority of the DEPs originated from the nucleus (n = 39, 32.5 %), cytoplasm (n = 21, 17.5 %), extracellular space (n = 20, 16.67 %), and plasma membrane (n = 19, 15.83 %) (Fig. 4A). Fig. 4B shows that in the 2 mM-VS-NC comparison, the majority of the DEPs originated from the nucleus (n = 86, 32.82 %), cytoplasm (n = 55, 20.99 %), and extracellular space (n = 39, 14.89 %).

The distribution of DEPs was further examined using the GO analysis tool. The biological functions were categorized into biological process, cellular component, and molecular function, resulting in the identification of 26 distinct groups for both 1 mM-VS-NC and 2 mM-VS-NC. In the biological process category, 99 and 75 DEPs were linked to cellular process and biological regulation, respectively, in 1 mM-VS-NC. Contrastingly, in 2 mM-VS-NC, 218 and 174 DEPs were connected to cellular process and biological regulation, respectively. In the cellular components category, the DEPs were predominantly linked with the cellular and organelle functions, with 105 and 90 DEPs, respectively, in 1 mM-VS-NC. Similarly, in 2 mM-VS-NC, the majority of DEPs were involved in cellular and organelle functions, with 232 and 203 DEPs, respectively. In 1 mM-VS-NC, the DEPs mainly demonstrated associations with binding and catalytic functions (75 and 41, respectively) within the molecular function category. Likewise, in 2 mM-VS-NC, the DEPs predominantly displayed connections with binding and catalytic functions (169 and 89, respectively) (Fig. 4C and D).

Additionally, the categorizations of DEPs were further analyzed based on the KOG database. In 1 mM-VS-NC, the findings highlighted that most DEPs were linked to protein turnover through posttranslational modification, chaperones, and signal transduction mechanisms, with 10 and 19 instances, respectively. In the 2 mM-VS-NC comparison, the DEPs were primarily involved in signal transduction mechanisms and transcription, with 29 and 27 proteins, respectively, associated with these functions (Fig. 4E and F).

### 3.5 Functional enrichment analysis of DEPs

**Fig. 5:**
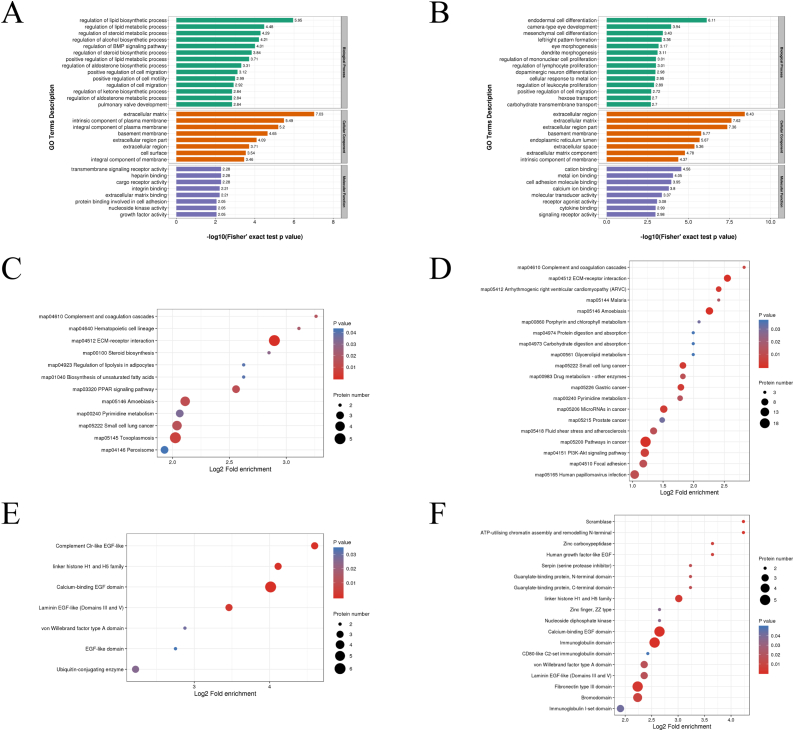
Functional enrichment analysis of DEPs. (A, B) GO-based enrichment analysis of DEPs in 1 mM-VS-NC and 2 mM-VS-NC, respectively. (C, D) The bubble charts of KEGG pathway analysis showed 12 and 20 signal pathways with the most significant enrichment in 1 mM-VS-NC and 2 mM-VS-NC, respectively. (E, F) The bubble charts of domain enrichment analysis showed 7 and 18 protein domains with the most significant enrichment in 1 mM-VS-NC and 2 mM-VS-NC, respectively.

To elucidate the role of DEPs, enrichment analysis was performed in each research subject using three levels of GO classification, KEGG pathway, and protein domain. Initially, GO annotation analysis was conducted, dividing 30 distinct groups into three categories: biological process (n = 14), cellular component (n = 8), and molecular function (n = 8) for both 1 mM-VS-NC and 2 mM-VS-NC. The analysis revealed that DEPs were significantly enriched in five identical categories, including the promotion of cell movement, components of the extracellular matrix (ECM), basement membrane, specific parts of the extracellular region, and the overall extracellular region (Fig. 5A and B).

Moreover, a KEGG pathway enrichment analysis was conducted. The results unveiled significant involvement of both comparison groups in enriched signal pathways, such as complement and coagulation cascades, ECM-receptor interaction, amoebiasis, pyrimidine metabolism, and small cell lung cancer (Fig. 5C and D).

**Fig. 6:**
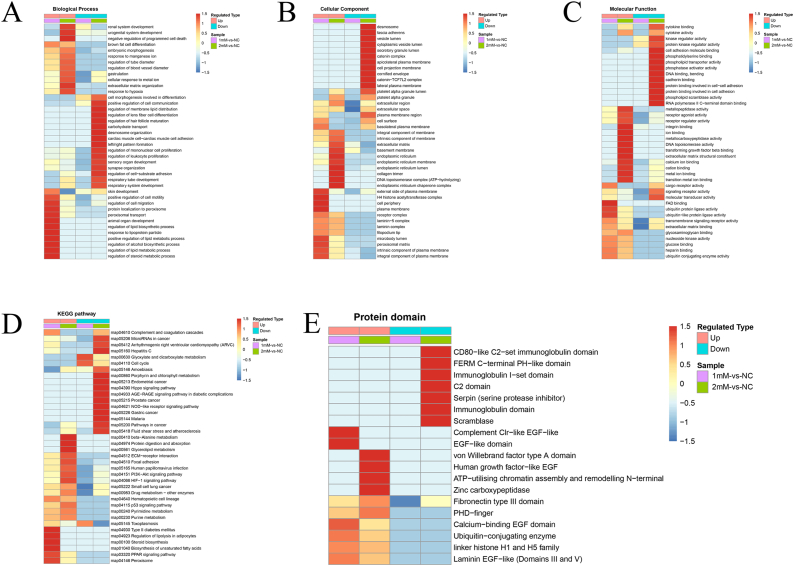
Hierarchical clustering analysis of DEPs. (A, B, C) Hierarchical clustering analysis of GO-based enrichment, including biological process, cellular component and molecular function. (D) Hierarchical clustering analysis of KEGG pathway. (E) Hierarchical clustering analysis of protein domain. The color bar indicates the degree of enrichment. Red indicates high enrichment and blue indicates weak enrichment.

Additionally, protein domain enrichment analysis was conducted to gain in-depth insight into the role of DEPs. The findings indicated that both 1 mM-VS-NC and 2 mM-VS-NC exhibited four identical protein domains, including the calcium-binding EGF domain, laminin EGF-like (Domains III and V), linker histone H1 and H5 family, and von Willebrand factor type A domain (Fig. 5E and F). Hierarchical clustering analysis (Fig. 6A–E) was used to visualize the functional enrichment analysis of DEPs across GO, KEGG pathways, and protein domains.

### 3.6 PRM validating target proteins

**Fig. 7:**
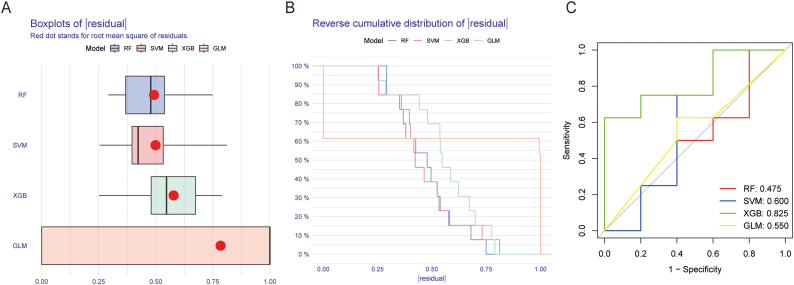
Construction and evaluation of XGB, SVM, RF and GLM machine models. (A) Boxplots showed the residuals of each machine learning model. Red dot represented the root mean square of residuals. (B) Cumulative residual distribution of each machine learning model. (C) ROC analysis of four machine learning models based on 5-fold cross-validation in the testing cohort.

**Fig. 8:**
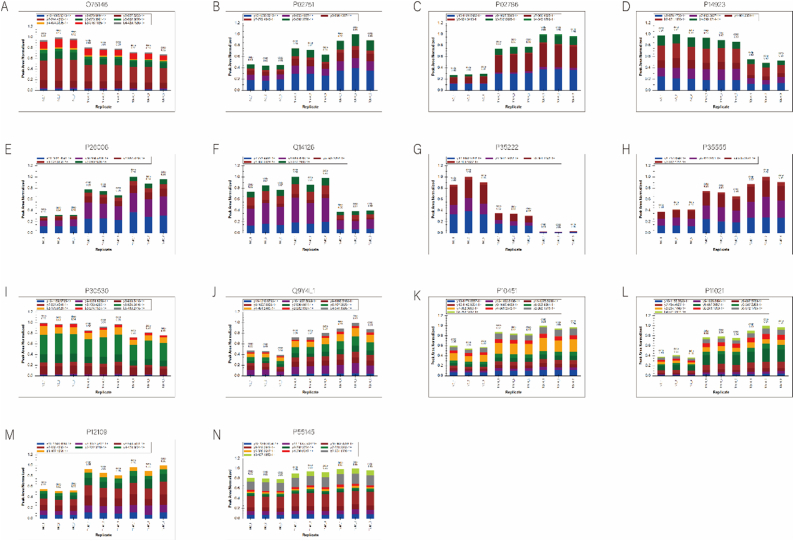
PRM validating target proteins. (A) The distribution diagram of fragment ion peak area of HIP1R (O75146). (B) The distribution diagram of fragment ion peak area of FN1 (P02751). (C) The distribution diagram of fragment ion peak area of TFRC (P02786). (D) The distribution diagram of fragment ion peak area of JUP (P14923). (E) The distribution diagram of fragment ion peak area of ITGA3 (P26006). (F) The distribution diagram of fragment ion peak area of DSG2 (Q14126). (G) The distribution diagram of fragment ion peak area of CTNNB1 (P35222). (H) The distribution diagram of fragment ion peak area of FBN1 (P35555). (I) The distribution diagram of fragment ion peak area of AXL (P30530). (J) The distribution diagram of fragment ion peak area of HYOU1 (Q9Y4L1). (K) The distribution diagram of fragment ion peak area of SPP1 (P10451). (L) The distribution diagram of fragment ion peak area of HSPA5 (P11021). (M) The distribution diagram of fragment ion peak area of COL6A1 (P12109). (N) The distribution diagram of fragment ion peak area of MANF (P55145). The height of column represents the level of protein expression.

Fig. 7 shows the identification of hub genes using GLM, RF, SVM-RFE, and XGboost algorithms. To validate the findings of the LC-MS/MS-based proteomic analysis in 1 mM-VS-NC and 2 mM-VS-NC, we chose 14 highly intriguing differentially expressed proteins (DEPs) in our research. According to Table 2, the PRM findings were consistent with the previous results regarding the alterations in levels of the target protein expression. Upregulation was observed in nine proteins, namely FN1, TFRC, ITGA3, FBN1, HYOU1, SPP1, HSPA5, COL6A1, and MANF, based on their corresponding gene names. The HIP1R, JUP, AXL, and CTNNB1 genes were responsible for the downregulation of four additional proteins. Furthermore, a sole protein labeled as DSG2 gene exhibited upregulation in the 1 mM CaOx intervention cohort while displaying downregulation in the 2 mM CaOx intervention cohort. The results were quantified by peak area from PRM. Fig. 7 displays the distribution diagram of the peak area of selected peptides in 9 samples for fragment ion (Fig. 7A–C). Despite the use of different methods, the fold-change values did not align with those obtained from LC-MS/MS-based proteomic analysis; however, the trend of DEPs remained consistent. To sum up, the results obtained from 4D-LFQ proteomic quantitative analysis were trustworthy (see Fig. 8).

**Table 2:** 14 DEPs of interest were selected to validate target proteins by PRM.

Protein Accession	Gene Name	1 mM-VS-NC fold-change	Regulated Type	2 mM-VS-NC fold-change	Regulated Type	2 mM-VS-1mM fold-change	Regulated Type
O75146	HIP1R	0.8136	Down	0.7217	Down	0.8870	Down
P02751	FN1	1.4889	Up	1.8572	Up	1.2473	Up
P02786	TFRC	2.7035	Up	3.4794	Up	1.2869	Up
P14923	JUP	0.9390	Down	0.5708	Down	0.6078	Down
P26006	ITGA3	2.4770	Up	3.0889	Up	1.2470	Up
P30530	AXL	0.9310	Down	0.7773	Down	0.8348	Down
P35222	CTNNB1	0.5294	Down	0.2116	Down	0.3997	Down
P35555	FBN1	1.7496	Up	2.2850	Up	1.3059	Up
Q14126	DSG2	1.2066	Up	0.4948	Down	0.4100	Down
Q9Y4L1	HYOU1	1.7721	Up	2.0743	Up	1.1705	Up
P10451	SPP1	1.4965	Up	1.7061	Up	1.1400	Up
P11021	HSPA5	1.9865	Up	2.3251	Up	1.1704	Up
P12109	COL6A1	1.6478	Up	1.8888	Up	1.1462	Up
P55145	MANF	1.1914	Up	1.3872	Up	1.1643	Up

## 4 Discussion

In the current investigation, the innovative proteomics technique 4D-LFQ was utilized to gain a more comprehensive understanding of the modified proteins in HK-2 cells exposed to varying CaOx concentrations. The 4D-LFQ method, an enhancement of the 3D-LFQ technique through the incorporation of a fourth dimension of ion mobility separation, enables the effective differentiation and detection of concealed protein signals with lower abundance. The 4D-LFQ proteomic quantitative technique also offers faster speed, higher sensitivity and accuracy, and greater flux [19]. According to the results of 4D-LFQ proteomic quantitative analysis, there were 120, 262, and 81 differentially expressed proteins (DEPs) in the 1 mM-VS-NC, 2 mM-VS-NC, and 2 mM-VS-1mM conditions, respectively. According to GO annotation, the functional enrichment analysis indicates that the differentially expressed proteins (DEPs) were notably enriched in promoting cell migration and the extracellular matrix, among other functions. Analysis of enrichment, based on the KEGG pathway, revealed significant enrichment of DEPs in complement and coagulation cascades, as well as in ECM-receptor (extracellular matrix-receptor) interaction and other related pathways. 14 DEPs of great interest were selected as candidate proteins, including FN1, TFRC, ITGA3, FBN1, HYOU1, SPP1, HSPA5, COL6A1, MANF, HIP1R, JUP, AXL, CTNNB1 and DSG2.The data from PRM demonstrated the variation trend of 14 DEPs was identical as 4D-LFQ proteomic quantitative analysis.Proteomics studies of CaOx-induced HK-2 cells using 4D-LFQ proteomic quantitative analysis and PRM may help to provide crucial potential target proteins and signaling pathways for elucidating the mechanism of nephrolithiasis and better treating nephrolithiasis.

Epidemiological data indicates that kidney stones affect approximately 1.7–14.8 % of the global population, with a recurrence rate exceeding 50 % within a five-year period, imposing a significant burden on the public and society [20]. Prior research has established that renal tubular epithelial cell damage significantly influences the initiation of kidney stone formation. Notably, hyperuricemia favors excessive oxidative stress and ROS formation, leading to renal tubular epithelial cell damage [21]. Elevated CaOx levels can induce endoplasmic reticulum stress (ERS), impaired autophagy, and ferroptosis in renal tubular epithelial cells, resulting in stone formation [22,23]. Nevertheless, the mechanism underlying kidney stone formation remains unclear. Despite significant advancements in surgical technology, progress in pharmacological treatments for renal stones remains limited [24,25]. Thus, the exploration of the mechanisms underlying nephrolithiasis, devising targeted therapies, and advances in proteomics are crucial. In the current study, the 4D-LFQ proteomic quantitative method was utilized for acquiring comprehensive data on protein modification in HK-2 cells subjected to varying CaOx levels.

The KEGG pathway analysis highlighted that the ECM-receptor interaction pathway was highly enriched in both the 1 mM-VS-NC and 2 mM-VS-NC samples. The ECM, comprised of an array of diverse large molecules, is vital in maintaining the structure and functionality of cells and tissues. Previous research has indicated that ECM-receptor interactions can directly or indirectly influence various cellular functions, including adhesion, migration, differentiation, proliferation, and apoptosis [26]. During the current investigation, several proteins, namely FN1, SPP1, COL6A1, and ITGA3, were identified as significant components in ECM-receptor interactions, thereby warranting further attention. The FN1 gene encodes fibronectin, a high-molecular-weight glycoprotein present in the ECM, known to interact with entities such as CD44, fibrin, integrins, heparan sulfate, and collagens [27]. FN1 participates in regulatory mechanisms encompassing multiple biological functions, playing pivotal roles in the adhesion, migration, and movement of diverse cells. Moreover, it is involved in blood coagulation, wound healing, and, noteworthily, in cancer progression [28,29]. According to Ji et al., excessive FN1 expression could hinder cell death and trigger the PI3K/Akt signaling pathway, thereby enhancing cell survival. Additionally, previous research has indicated that stones contained FN1 in their protein matrix [30]. Supaporn et al. reported that FN1 could exhibit a dual role in the interaction between renal tubular epithelial cells and CaOx crystals [31]. FN1 not only inhibits COM crystallization, crystal growth, and adhesion to renal tubular cells, but it also promotes COM crystal aggregation and invasion through ECM. Interestingly, autophagy may contribute to the accumulation of FN1 in proximal tubular cells [32]. Atsushi et al. demonstrated that FN1 could act as a regulatory factor for macrophage phagocytosis [27], a process critical for crystal elimination. Integrin alpha-3, encoded by ITGA3 and part of the integrin family, functions as a cellular adhesion molecule on the cell surface, facilitating interaction with proteins in the ECM [33]. ITGA3 can mediate cell adhesion and proliferation and maintain cell morphology by connecting with the ECM [34]. ITGA3, a key transmembrane protein, plays a pivotal role in the upstream regulation of the P13K-AKT signaling pathway due to its function in signal transduction [35]. Notably, in the KEGG analysis, the four proteins involved in ECM-receptor interaction, namely FN1, SPP1, COL6A1, and ITGA3, are also implicated in the PI3K-AKT signaling pathway, which is crucial in cell growth and viability [36,37]. Prior research has demonstrated that the PI3K-AKT signaling pathway is essential in controlling cell growth, maintaining metabolic balance, preventing cell death, and regulating various cellular functions [[38], [39], [40]]. Wang et al. demonstrated that inhibition of the PI3K/Akt signaling pathway could reduce the progression of EMT, inhibit the apoptosis of renal tubular epithelial cells, and subsequently hinder the formation and expansion of CaOx crystals. In contrast, Liu et al. [40] discovered that the activation of the Akt pathway could enhance cell proliferation and provide protection against apoptosis in HK-2 cells exposed to CaOx crystals. Noteworthily, Yu et al. [39] documented that the activated PI3K/Akt signaling pathway could increase p38 MAPK levels, leading to the disruption of tight junctions in renal tubular epithelial cells when exposed to COM crystals. In the current research, all four proteins exhibited upregulation in 1 mM-VS-NC and 2 mM-VS-NC, with the extent of upregulation differing based on the CaOx concentration. Only a limited number of studies have reported the correlation between these molecules or proteins in the PI3K/AKT signaling pathway and the mechanism underlying urolithiasis. Hence, establishing a connection between ECM-receptor interactions and the PI3K/Akt signaling pathway may yield profound insights into the pathogenesis of nephrolithiasis. Interestingly, several studies have posited FN1 and ITGA3 as potential autonomous prognostic biomarkers for various diseases, including ovarian cancer, pancreatic cancer, oral squamous cell carcinoma, and breast cancer [33,41,42]. Nonetheless, further investigation is warranted to ascertain whether these target proteins can serve as predictive markers for urolithiasis, especially given that the levels of OPN, encoded by SPP1, may play a valuable role in assessing the prognosis and treatment of urolithiasis in both urine and serum [43].

HSPA5, also known as GRP78 or BiP, is a chaperone located within the endoplasmic reticulum (ER) and belongs to the HSP70 family of Heat Shock Proteins. GRP78, present in the ER membrane of all eukaryotes, acts as a central controller in preserving cellular equilibrium amidst ER stress through the initiation of unfolded protein response (UPR) [9,[44], [45], [46]]. In the current research, exposure to CaOx led to a marked increase in the upregulation of GRP78. In addition, the expression of GRP78 exhibited a significant dose-dependent upregulation when comparing 1 mM-VS-NC and 2 mM-VS-NC. The increase in GRP78 levels acts as a signal for ER stress, which is directly correlated with ER function [44,46]. Hence, it was hypothesized that the presence of CaOx stimulation could lead to unfolded protein accumulation within the ER of HK-2 cells and the subsequent release of GRP78 from the sensors of the UPR, activating the UPR to maintain ER homeostasis. However, prolonged ER stress eventually could result in irreversible cell injury and death. The initiation of ER stress by CaOx through GRP78 upregulation at the protein level was briefly illustrated. Furthermore, Bi et al. [45] proposed that GRP78 could engage with caspase 7 or Bcl-2, thereby repressing apoptosis. The activation of the Akt pathway by GRP78 to counteract oxidative stress and control Raf-1 within mitochondria, ultimately protecting cells against apoptosis, has also been suggested. HYOU1, also known as GRP170, is widely acknowledged as a protein indicative of UPR activation [47]. The HYOU1 protein showed a notable increase in 1 mM-VS-NC and a further elevation in 2 mM-VS-NC during our investigation. Several experiments have indicated that HYOU1 could have a fundamental cytoprotective role in cellular perturbation under hypoxia and could promote cell survival under ER stress [48,49]. According to Dominique et al. [49], the HYOU1 protein was strongly linked to the HSPA5 protein, collaborating to preserve protein homeostasis. Additionally, the current research demonstrated that the Mesencephalic Astrocyte-derived Neurotrophic Factor (MANF) protein levels were slightly increased in 1 mM-VS-NC, while significantly elevated in 2 mM-VS-NC. MANF, highly responsive to ER stress and abundantly present in various tissues, belongs to a group of proteins that are the most susceptible to ER stress [50]. Numerous studies have uncovered that enhanced MANF expression can have a protective effect on cells and prevent cell death by alleviating ER stress [51,52]. A previous study has reported that MANF could protect liver cells against damage through the PI3K/Akt/GSK3β pathway [50]. Intriguingly, Eesmaa et al. [53] concluded that MANF could serve as a co-factor of GRP78 to regulate UPR and maintain protein homeostasis. The role of MANF in kidney diseases, however, remains elusive. The UPR aims to restore the normal function of ER5 by addressing inflammatory responses, damage, and oxidative stress, which are closely associated with ERS. Nonetheless, these adaptive responses may eventually be overwhelmed by the ER stress, generating a proapoptotic response [5]. Various studies have confirmed that ER stress is crucial in the development of kidney stones [46,54]. The present investigation has revealed that the HYOU1, HSPA5, and MANF proteins were elevated in HK-2 cells when exposed to CaOx, suggesting the presence of ER stress in these cells, potentially leading to apoptosis. Furthermore, Abdo et al. [55] have reported that directing efforts towards GRP78 could yield an innovative approach to engage in the fight against cancer and inhibit the growth of fungi and viruses. Prior research has also indicated that GRP170 could be employed for the development of specific chaperone vaccines to address metastatic cancers, while MANF could be a promising therapeutic biomarker for ERS-related diseases [56,57]. Overall, studying HYOU1, HSPA5, and MANF could provide deeper insight into the role of ER stress in the pathogenesis of urolithiasis and their potential as novel targeted therapeutic approaches for nephrolithiasis.

Transferrin receptor 1 (TfR1/CD71/TFRC) is a transmembrane glycoprotein that binds with transferrin (Tf) on the cell membrane to internalize the iron bound to Tf through endocytosis [23,58]. Cellular iron acquisition is regulated by TfR1 expression [59]. According to Christopher et al. [60], kidneys exhibited the highest TfR1 levels compared to other organs, with TfR1 primarily localized in the basal membrane and cytoplasm of the renal tubular epithelium. Furthermore, according to Craig et al. [61], the expression of TfR1 was most prominent in the proximal tubule, decreasing progressively along the tubule. In the current study, a significant rise in TFRC expression was noted in 1 mM-VS-NC and an even greater elevation in 2 mM-VS-NC. Several studies have indicated that TfR1 could be vital in preserving cellular iron balance [62,63]. Previous findings also suggested that excessive accumulation of Fe^2+^ within cells could be essential in triggering ferroptosis, which is closely associated with the production of ROS, autophagy, and ERS in HK-2 cells when exposed to CaOx [23]. Consequently, it was proposed that TfR1 level increase could heighten Tf binding when renal tubule epithelium cells are subjected to hyperoxaluria, ultimately leading to excessive intracellular Fe^2+^ and ferroptosis.

Fibrillin-1 (FBN-1), a prevalent ECM protein, is involved in cellular repair and matrix restructuring [64]. Li et al. [65] discovered that elevated FBN1 levels in the extracellular environment could induce endothelial cell damage and programmed cell death and inhibit their growth by activating the integrin v6/TGF-/Smad3 signaling pathway. FBN1-enriched extracellular microenvironments were implicated in mediating cross-talks between peritubular capillary endothelium and injured tubules, inducing apoptosis and capillary integrity loss. Peng et al. [66] demonstrated the potential of plasma FBN1 as a reliable biomarker for diagnosing spontaneous coronary artery dissection. In the current study, FBN-1 seemed to be regulated in both 1 mM-VS-NC and 2 mM-VS-NC, necessitating further investigation to determine the role of FBN-1 in causing renal tubular epithelial cell injury. Differential expression of AXL, HIP1R, JUP, CTNNB1, and DSG2 was confirmed via PRM, suggesting their potential roles in the development and advancement of nephrolithiasis, warranting further exploration.

Future research in nephrolithiasis is expected to leverage proteomics application across various facets, involving multidisciplinary collaboration among scientists, nephrologists, urologists, and researchers to identify proteins and their interactions with crystals. The focus will be to deepen the understanding of crystal-protein dynamics and potentially discover novel urinary stone formation inhibitors or promoters. Proteomic methods are expected to serve as initial screening tools, followed by functional validation through conventional biochemical techniques. An emerging area of interest, previously unexplored via proteomics, is the study of cellular responses during crystal adhesion to renal tubular epithelial cells.

Proteomics offers a wealth of data and opportunities in kidney stone research. The primary goal is to enhance comprehension of the etiology and molecular mechanisms underlying stone formation, identify biomarkers for early detection and accurate prediction of stone recurrence, and discover novel therapeutic targets to improve treatment outcomes and efficiently prevent kidney stones.

## 5 Conclusion

In an endeavor to provide a comprehensive overview of the proteins implicated in the development of kidney stones, 4D-LFQ proteomic quantitative methods were employed. This research presents a spectrum of potentially valuable target proteins and signaling pathways, offering deeper insights into the mechanism of nephrolithiasis. Such insights could enhance the accuracy of diagnostic processes and the efficacy of pharmacological interventions. Nevertheless, acknowledging a notable limitation in the present investigation is crucial. The in vitro conditions, specifically the CaOx concentrations used, do not entirely mirror the in vivo environment wherein renal tubule epithelial cells are exposed to urine oversaturated with CaOx. Future research endeavors must be directed toward designing and implementing more comprehensive experiments to further enrich and clarify the findings of this study.

## Disclosure statement

There are no conflicts of interest among the authors.

## Funding

The National 10.13039/501100001809Natural Science Foundation of China supported this research (No. 82070723; No. 82270797).

## Data availability

The data that has been used is confidential.

## CRediT authorship contribution statement

**Ziqi He:** Data curation, Conceptualization. **Jiawei Zhou:** Writing – original draft, Formal analysis, Data curation. **Caitong Dong:** Writing – review & editing, Visualization, Software. **Chao Song:** Writing – review & editing, Visualization, Validation. **Wenbiao Liao:** Project administration, Methodology, Investigation. **Yunhe Xiong:** Writing – review & editing, Supervision, Project administration. **Sixing Yang:** Writing – review & editing, Supervision, Funding acquisition.

## Declaration of competing interest

The authors declare that they have no known competing financial interests or personal relationships that could have appeared to influence the work reported in this paper.
